# The efficacy and safety of ginkgo terpene lactone preparations combined with antiplatelet aents in the treatment of ischemic stroke: a systematic review and meta-analysis

**DOI:** 10.3389/fphar.2025.1554207

**Published:** 2025-03-19

**Authors:** Hong Xu, Li Zeng, Li Liao, Xiaoxuan Li, Yan Tang

**Affiliations:** ^1^ Department of Pharmacy, The Second People’s Hospital of Yibin, Yibin, China; ^2^ Department of Pharmacy, The Third People’s Hospital of Yibin, Yibin, China

**Keywords:** ischemic stroke, DGMI, ginkgolide injection, antiplatelet drugs, meta-analysis

## Abstract

**Background:**

This meta-analysis aimed to assess the efficacy and safety of ginkgo terpene lactone preparations including diterpene ginkgolides meglumine injection (DGMI) and ginkgolide injection combined with antiplatelet drugs in the treatment of ischemic stroke.

**Methods:**

We systematically searched the randomized controlled trials(RCTs) with publication date earlier than 6 November 2024 in PubMed, China National Knowledge Infrastructure (CNKI), Chinese Science and Technology Journal Database (VIP), Chinese Biomedical Literature Database (CBM), Wanfang Database, Embase, Web of Science, ClinicalTrials.gov, and Cochrane Library. Studies were screened according to inclusion and exclusion criteria, evaluated according to criteria recommended by the Cochrane Handbook, and data were then analyzed using Stata 17 software.

**Results:**

Of 1,079 identified studies, 27 were eligible and included in our analysis (N = 3,336 patients). The meta-analysis demonstrated that the overall response rate [RR = 1.22, 95% CI(1.17, 1.27), Z = 9.76, *p* < 0.01], as well as the National Institutes of Health Stroke Scale (NIHSS) score and barthel index, were significantly better in the DGMI combined treatment group compared to the antiplatelet therapy alone group. However, there was no significant difference observed between the experimental group and the control group regarding improvements in prognosis and platelet function. The studies included in the analysis reported a total of 419 adverse reactions (ADRs), with 206 occurring in the DGMI combined treatment group; furthermore, there was no significant difference in the incidence of adverse events between the two groups.

**Conclusion:**

Ginkgo terpene lactone preparations, when combined with antiplatelet drugs, can significantly enhance the clinical efficacy of ischemic stroke and demonstrate a favorable safety profile. This combination is a potential treatment strategy that can improve the management of IS patients and has high clinical application value.

## Introduction

Ischemic stroke is a significant cause of disability and mortality worldwide. Data from the Global Burden of Disease study indicate that ischemic stroke impacts millions of patients annually, imposing significant financial and emotional burdens on individuals, families, and society at large (VL et al., 2018; GBD, 2019 Stroke Collaborators, 2021). Currently, the primary treatments for ischemic stroke include thrombolytic therapy and antiplatelet therapy (Powers et al., 2019). While thrombolytic therapy can effectively reopen occluded vessels in patients with ischemic stroke, it is constrained by a strict time window for administration and carries risks, such as bleeding (Tsivgoulis et al., 2023). Antiplatelet drugs, such as aspirin, clopidogrel, and tirofiban, are crucial for the secondary prevention of ischemic stroke. However, the efficacy of these single agents in enhancing long-term prognosis is limited, and issues such as drug resistance persist (Kamarova et al., 2022). Consequently, there is an urgent need to investigate and validate novel treatment combinations to enhance recovery following a stroke.

Ginkgo terpene lactones represent a class of compounds derived from the leaves of Ginkgo biloba, primarily comprising bilobalide and ginkgolides A, B, C, K, J, L, M, N, P, and Q (Geng et al., 2018) Previous studies have established that related preparations, such as DGMI and ginkgolide injections, exhibit significant efficacy in treating cerebrovascular diseases (Zhao et al., 2022; Li et al., 2024). To be specific, DGMI is composed of ginkgolide A, ginkgolide B, and ginkgolide K. ginkgolide injection contains bilobalide, ginkgolide A, ginkgolide B, and ginkgolide C. In recent years, the therapeutic strategy involving ginkgo terpene lactone preparations with antiplatelet drugs has garnered significant attention within the academic community. The theoretical advantages of this combined treatment regimen are multifaceted. Firstly, ginkgo terpene lactones provide neuroprotection through antioxidant and anti-inflammatory mechanisms, which complement the effects of antiplatelet drugs and may enhance overall therapeutic efficacy (Xia and Fang, 2007). Secondly, ginkgo terpene lactones facilitate an increase in oxygen supply to ischemic regions by augmenting cerebral blood flow and improving microcirculation, thereby promoting recovery (Xu et al., 2005; Zhu et al., 2022). Lastly, ginkgo terpene lactones, as platelet-activating factor receptor (PAFR) antagonists, may synergize with conventional antiplatelet regimens in antithrombotic treatment by inhibiting platelet aggregation (Han et al., 2023). The synergistic effects of these multiple pathways position this approach as a promising strategy for enhancing clinical outcomes.

Based on the aforementioned rationale, numerous clinical trials have evaluated the efficacy and safety of ginkgo terpene lactone preparations in conjunction with antiplatelet agents. However, there remains a notable absence of systematic reviews and meta-analyses that provide a comprehensive assessment of their actual efficacy and safety in clinical practice. Therefore, we conducted a systematic review and meta-analysis of existing randomized controlled trials to determine the effect of this combination regimen on key outcomes such as clinical efficacy, NIHSS score, platelet function, and safety in patients with ischemic stroke.

## Materials and methods

### Searching strategy

This systematic review and meta-analysis is reported in accordance with the Preferred Reporting Items for Systematic Reviews and Meta-Analyses (PRISMA) Statement. The protocol for this systematic review was registered on INPLASY(Unique ID number is INPLASY2024120106) and is available in full on inplasy.com (https://doi.org/10.37766/inplasy2024.12.0106). We systematically searched PubMed, CNKI, VIP, CBM, Wanfang Database, Embase, Web of Science, ClinicalTrials.gov, and Cochrane Library. The search ranged from the time each database was built to 6 November 2024. Screening language is Chinese or English. We used the following combined textword and MeSH terms such as “Ischemic Stroke,” “Ginkgolide” and “ginkgo diterpene lactone meglumine”. The complete search used for PubMed was: ((“Ischemic Stroke” [Mesh]) OR (Stroke*[Title/Abstract])) AND ((“Ginkgolides” [Mesh]) OR ((((Ginkgolide*[Title/Abstract]) OR (ginkgo diterpene lactone meglumine [Title/Abstract])) OR (GDLM [Title/Abstract])) OR (DGLM [Title/Abstract]))).

### Inclusion criteria

Inclusion criteria were abided by the participants, interventions, comparison/control, outcomes, and study design (PICOS) format, which were as follows: 1) The study participants included adults aged 18 years and older, irrespective of gender or ethnicity. Ischemic stroke was diagnosed using computed tomography (CT) or magnetic resonance imaging (MRI), in accordance with established diagnostic criteria. 2) Study design: RCTs in Chinese or English. 3) Intervention measures: Both the experimental group and the control group received conventional treatment, which encompasses the regulation of blood lipids, oxygen inhalation, blood pressure control, blood glucose regulation, and nutritional supplementation. In addition to conventional treatment, the experimental group was administered ginkgo terpene lactone preparations in combination with antiplatelet drugs. The ginkgo terpene lactone preparations included DGMI and ginkgolide injection. 4) The control group received only antiplatelet drugs on the basis of conventional treatment. 5) The outcome measures met the following primary or secondary outcomes: the primary outcomes were the clinical efficacy defined according to the nationally approved criteria (The Fourth National Conference on cerebrovascular. 1996; Wang, 2024; Chen et al., 2022). Specifically, reductions in neurological deficit scores ranging from 18% to 100% following treatment were classified as demonstrating varying degrees of clinical efficacy. Conversely, a decrease in neurological deficit score of less than 17% or an increase in score following treatment was deemed “ineffective.” As secondary outcomes, we compared the NIHSS score, Barthel index, modified Rankin scale (mRS), platelet function, and the incidence of ADRs or adverse events.

### Exclusion criteria

The exclusion criteria were as follows: 1) nonclinical randomized controlled trials, including literature reviews, systematic reviews, individual case studies, experience summaries, and basic research; 2) repeated publications; 3) imprecise descriptions of conventional treatment in both the experimental and control groups; 4) studies involving patients treated with thrombolytics or other neuroprotective agents (e.g., edaravone, citicoline); and 5) studies focused on other diseases.

### Data extraction and quality assessment

Two independent investigators (HX and YT) reviewed study titles and abstracts, and studies that satisfied the inclusion criteria were retrieved for full-text assessment. In case of disagreement, decisions were made by discussion or consultation with a third investigator (LZ). We extracted the relevant data of the included studies, and the extracted contents included: basic information of the included studies (title, first author, publication year, etc.); basic characteristics of the study subjects (number of patients, gender, age, intervention measures, dose, treatment process, etc.); outcome evaluation indicators; and risk of bias assessment information. Quality assessment was performed by the Cochrane risk of bias tool, including randomization method, allocation concealment, blinding, baseline comparability, and completeness of outcome data.

### Statistical analysis

Statistical analysis was conducted using Stata 17 software. Hazard ratios (RR) and standardized mean differences (SMD) served as effect indicators for dichotomous data and continuous variables, respectively. A 95% confidence interval (95% CI) was calculated for both types of data. The I^2^ test was employed to assess the heterogeneity among the included studies. If *p* > 0.1 and I^2^ < 50%, it was concluded that there was no heterogeneity among the studies, and a fixed-effect model was selected for analysis. Conversely, in cases of substantial heterogeneity, a random-effects model was utilized, and a sensitivity analysis was conducted to identify the sources of heterogeneity. Finally, funnel plots were generated to evaluate the publication bias present in the literature.

## Results

### Study selection process

**FIGURE 1:**
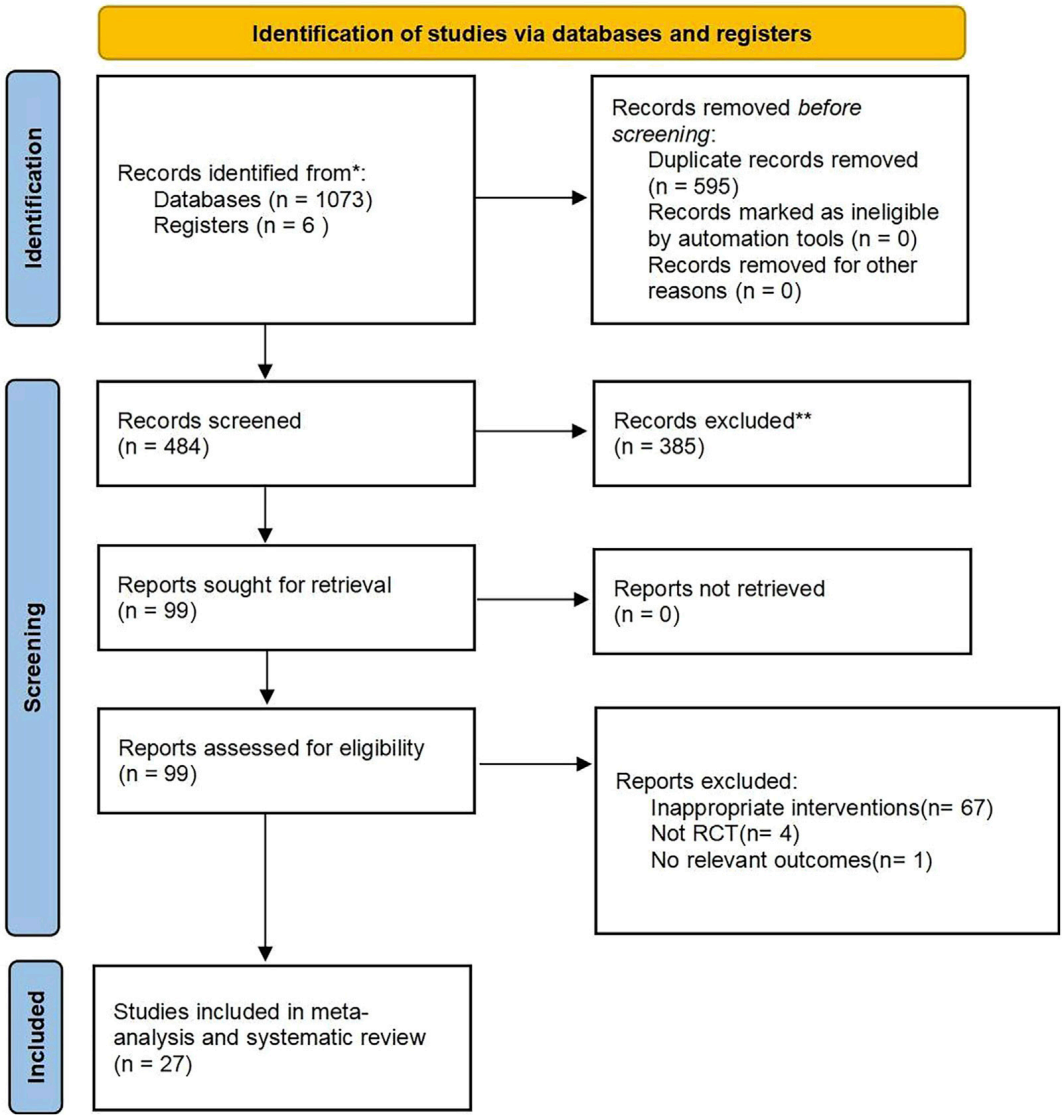
PRISMA flow diagram.

According to the search strategy, a total of 1,079 citations were retrieved. After the duplicate articles were removed, the remaining 484 articles were filtered according to the inclusion and exclusion criteria, and then 385 articles were excluded. By further reading the full text, finally 27 studies were included for meta-analysis. The screening process is shown in Figure 1.

### Study characteristics

The 27 included trials were published from 2017 to 2024, four (Chen et al., 2023; Wang, 2024; Dong et al., 2021; Chen et al., 2022) of which were published in English and the remaining trials were published in Chinese. A total of 3,336 patients were involved in this review, including 1,663 patients in the experimental group and 1,673 patients in the control group. Across all studies, the average age of participants was approximately 52–70 years, with more male participants than female participants. Antiplatelet agents used in the two included trials were mainly aspirin enteric-coated tablets, clopidogrel sulfate tablets, and ozagrel sodium for injection. The experimental group was treated with DGMI(25 mg, once a day) and ginkgolide injection (25 mg or 50 mg, once a day) for 14 days. Detailed characteristics of the included trials are detailed in Table 1.

**TABLE 1:** Basic characteristics of included studies.

Study ID	Cases		Sex (M/F)		Average age		NIHSS score before treatment		Intervention measures		Outcome
	E	C	E	C	E	C	E	C	E	C	
Chen et al. (2023)	35	35	29/6	21/14	70.40 ± 9.88	70.97 ± 11.19	10.05 ± 2.72	9.77 ± 2.18	DGMI + aspirin	placebo + aspirin	②⑤⑥
Dong et al. (2021)	463	473	283/180	316/157	64.31 ± 10.68	64.12 ± 10.40	6.22 ± 3.18	5.87 ± 2.50	ginkgolide injection + aspirin	placebo + aspirin	④⑥
Wang, (2024)	50	50	30/20	29/21	68.45 ± 5.17	69.53 ± 7.86	7.92 ± 2.28	8.06 ± 2.33	DGMI + aspirin and clopidogrel	aspirin and clopidogrel	①②⑥
Chen et al. (2022)	40	40	22/18	19/21	62.0 ± 14.3	62.5 ± 13.8	13.95 ± 4.32	14.25 ± 4.48	DGMI + aspirin and clopidogrel	aspirin and clopidogrel	①②③
Zhang, (2020)	36	36	29/7	24/12	66.71 ± 10.05	67.48 ± 9.79	11.28 ± 2.53	11.19 ± 2.71	DGMI + aspirin	aspirin	①②④⑥
Cai, (2022)	35	35	22/13	21/14	62.97 ± 4.46	62.86 ± 4.36	17.02 ± 1.97	16.99 ± 1.95	DGMI + ozagrel	ozagrel	①②
ZhangBo, (2020)	45	45	29/16	27/18	62.32 ± 9.23	61.21 ± 8.67	12.45 ± 3.12	11.96 ± 2.89	DGMI + clopidogrel	clopidogrel	①②③⑥
Zeng et al. (2020)	34	34	22/12	20/14	53.21 ± 6.68	52.03 ± 7.10	14.45 ± 2.03	14.14 ± 1.89	DGMI + aspirin	aspirin	①⑥
Zhang Q. et al. (2021)	30	30	16/14	15/15	57.57 ± 9.35	59.72 ± 9.73	—	—	DGMI + aspirin and clopidogrel	aspirin and clopidogrel	①⑤⑥
Shen et al. (2022)	46	46	26/20	24/22	58.63 ± 5.43	57.90 ± 5.13	—	—	DGMI + aspirin and clopidogrel	aspirin and clopidogrel	⑤
Song, (2022)	49	49	24/25	24/25	56.49 ± 6.31	57.29 ± 5.77	11.42 ± 3.37	11.28 ± 3.19	DGMI + ozagrel	ozagrel	①②③⑥
Shi, (2022)	37	37	20/17	22/15	60.48 ± 1.25	60.79 ± 1.30	28.49 ± 3.46	28.33 ± 3.12	DGMI + clopidogrel	clopidogrel	①②⑥
Guo, (2022)	43	43	28/15	27/16	58.50 ± 11.50	58.50 ± 10.50	16.37 ± 4.18	16.42 ± 4.12	DGMI + aspirin	aspirin	①②③⑥
Wang et al. (2024)	85	85	51/34	56/29	62.35 ± 7.18	61.40 ± 7.46	20.85 ± 3.76	20.17 ± 3.52	DGMI + aspirin and clopidogrel	aspirin and clopidogrel	①②③④⑥
Li et al. (2023)	52	52	23/29	29/23	64.01 ± 6.52	62.86 ± 6.39	23.01 ± 4.19	22.84 ± 4.27	DGMI + clopidogrel	clopidogrel	①②③⑥
Zheng et al. (2023)	30	30	18/12	16/14	68.69 ± 1.14	66.75 ± 1.28	4.36 ± 0.63	4.42 ± 0.55	DGMI + aspirin and clopidogrel	aspirin and clopidogrel	①②⑥
Li et al. (2022)	35	35	—	—	—	—	7.92 ± 3.64	8.52 ± 3.01	DGMI + aspirin	aspirin	①②
Guan, (2019)	43	43	28/15	25/18	57.2 ± 3.7	57.8 ± 3.5	—	—	ginkgolide injection + aspirin	aspirin	①③
Liu, (2019)	72	72	41/31	40/32	62.25 ± 4.77	62.18 ± 4.07	15.30 ± 1.02	15.28 ± 1.00	ginkgolide injection + aspirin	aspirin	①②③
Cui et al. (2018)	41	41	28/13	27/14	59.83 ± 5.47	59.47 ± 5.69	—	—	ginkgolide injection + aspirin	aspirin	①⑥
Yang. et al. (2018)	80	80	50/30	51/29	63.3 ± 3.8	61.9 ± 3.6	18.74 ± 3.51	18.69 ± 3.64	ginkgolide injection + aspirin and clopidogrel	aspirin and clopidogrel	①②③⑥
Chen Y. et al. (2018)	24	24	—	—	58 ± 6.22	60 ± 3.21	—	—	ginkgolide injection + aspirin	aspirin	①
Lei et al. (2017)	48	48	27/21	26/22	68.7 ± 4.5	69.3 ± 4.8	18.57 ± 3.46	18.38 ± 3.72	ginkgolide injection + aspirin	aspirin	①②③
Zhao, (2021)	55	55	28/27	30/25	68.32 ± 3.41	68.19 ± 3.27	18.50 ± 2.04	18.42 ± 1.95	ginkgolide injection + aspirin and clopidogrel	aspirin and clopidogrel	②⑥
Li et al. (2021)	67	67	43/24	41/26	62.37 ± 9.37	63.19 ± 10.65	-	-	ginkgolide injection + clopidogrel	clopidogrel	①
Sun et al. (2019)	36	36	18/18	20/16	62.54 ± 3.98	60.18 ± 4.33	11.02 ± 3.51	10.89 ± 3.27	ginkgolide injection + aspirin	aspirin	②⑥
Yang et al. (2019)	52	52	27/25	28/26	65.87 ± 7.63	63.52 ± 8.51	10.63 ± 2.51	10.07 ± 2.32	ginkgolide injection + aspirin	aspirin	②

Note: E, experimental group; C, control group; F, females, M, males. ①, clinical efficacy; ②, NIHSS, score; ③, Barthel index; ④, mRS, score; ⑤, Platelet function (AA-MAR, and ADP-MAR); ⑥, ADRs/ADEs, adverse drug reactions/adverse drug events.

### Quality evaluation on included studies

**FIGURE 2:**
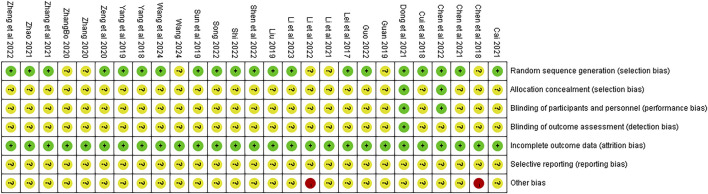
Quality evaluation of included literature.

The quality of the 27 studies is illustrated in Figure 2. Although all articles referenced “randomization,” only 20 studies (Chen et al., 2023; Liu, 2019; Lei et al., 2017; Shi, 2022; Cai, 2022; Zeng et al., 2020, Song 2022; Zheng et al., 2023; Sun et al., 2019; Yang et al., 2019; Dong et al., 2021; Yang. et al., 2018; Zhao, 2021, Zhang L. et al., 2021; Shen et al., 2022; Cui et al., 2018; Li et al., 2023; Wang et al., 2024; Guo, 2022; Chen et al., 2022) detailed specific randomization methods. Two studies (Chen et al., 2023; Dong et al., 2021) implemented double-blinding and allocation concealment through computer coding or third-party drafting protocols during the experiment; one (Dong et al., 2021) of these also conducted blinding in outcome assessments. While all articles provided complete outcome data, it remained unclear whether the authors selectively reported relevant results at the time of publication. Lastly, two studies (Li et al., 2022, Chen Y. et al., 2018) were classified as “high risk” due to incomplete baseline data, while the other studies did not disclose any additional risk deviations.

### Meta-analysis results

#### Analysis of clinical efficacy

**FIGURE 3:**
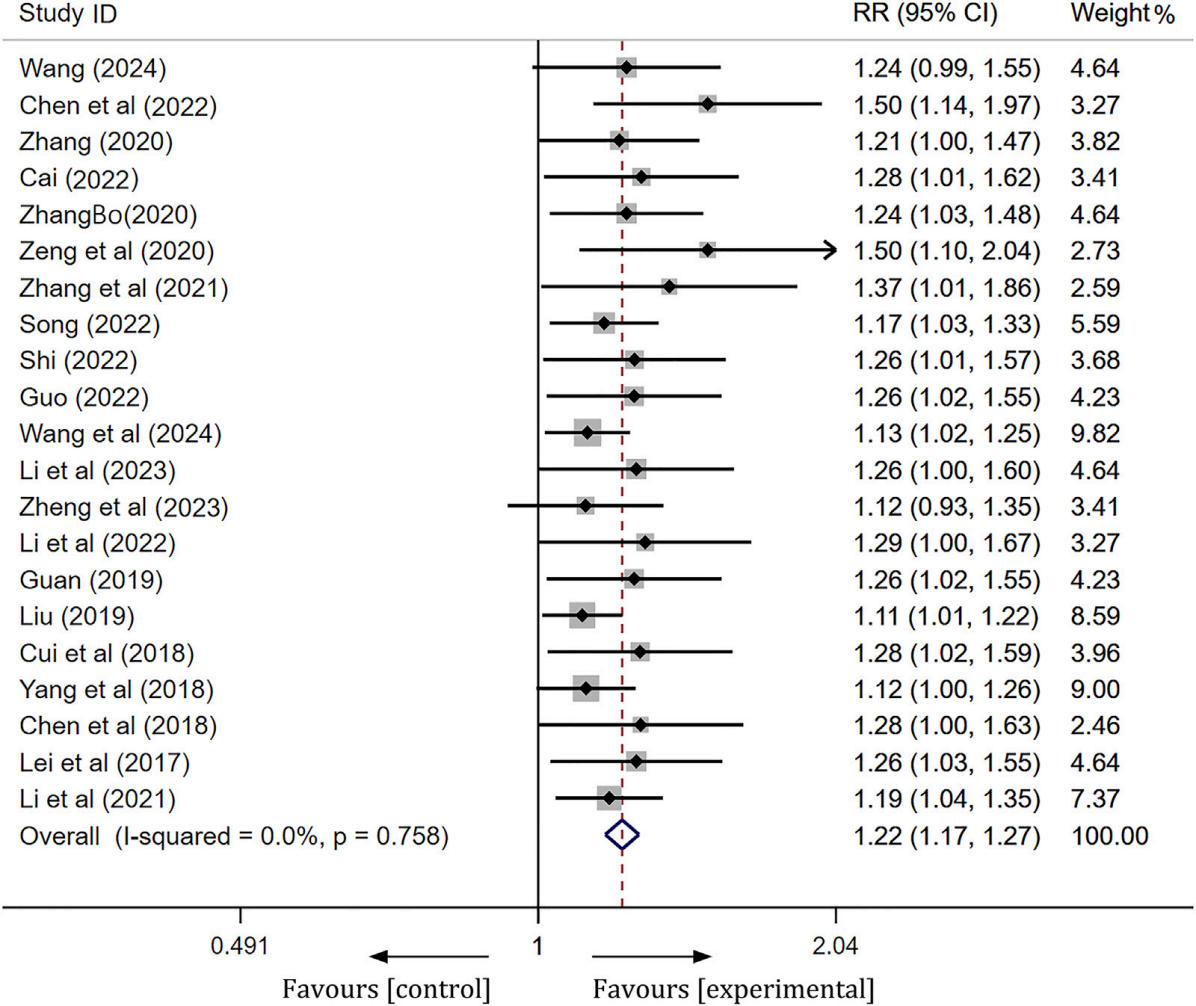
Forest plot of ginkgo terpene lactone preparations on clinical efficacy.

A total of 21 articles were compared regarding the clinical efficacy between the ginkgo terpene lactone preparations combined treatment group and the control group. Following a heterogeneity test, these 21 studies demonstrated no statistical heterogeneity (I^2^ = 0%, *p* = 0.758), thus permitting the application of a fixed effect model for analysis. To assess the accuracy and stability of the clinical efficacy derived from the meta-analysis, we conducted a sensitivity analysis utilizing a row-by-row exclusion method. As illustrated in Supplementary Figure S1, no significant interference was detected among the studies, indicating that the results were relatively stable. The meta-analysis revealed a statistically significant difference [RR = 1.22, 95% CI (1.17, 1.27), Z = 9.76, *p* < 0.01], suggesting that the clinical efficacy of the experimental group was significantly superior to that of the control group (Figure 3).

#### NIHSS scores

**FIGURE 4:**
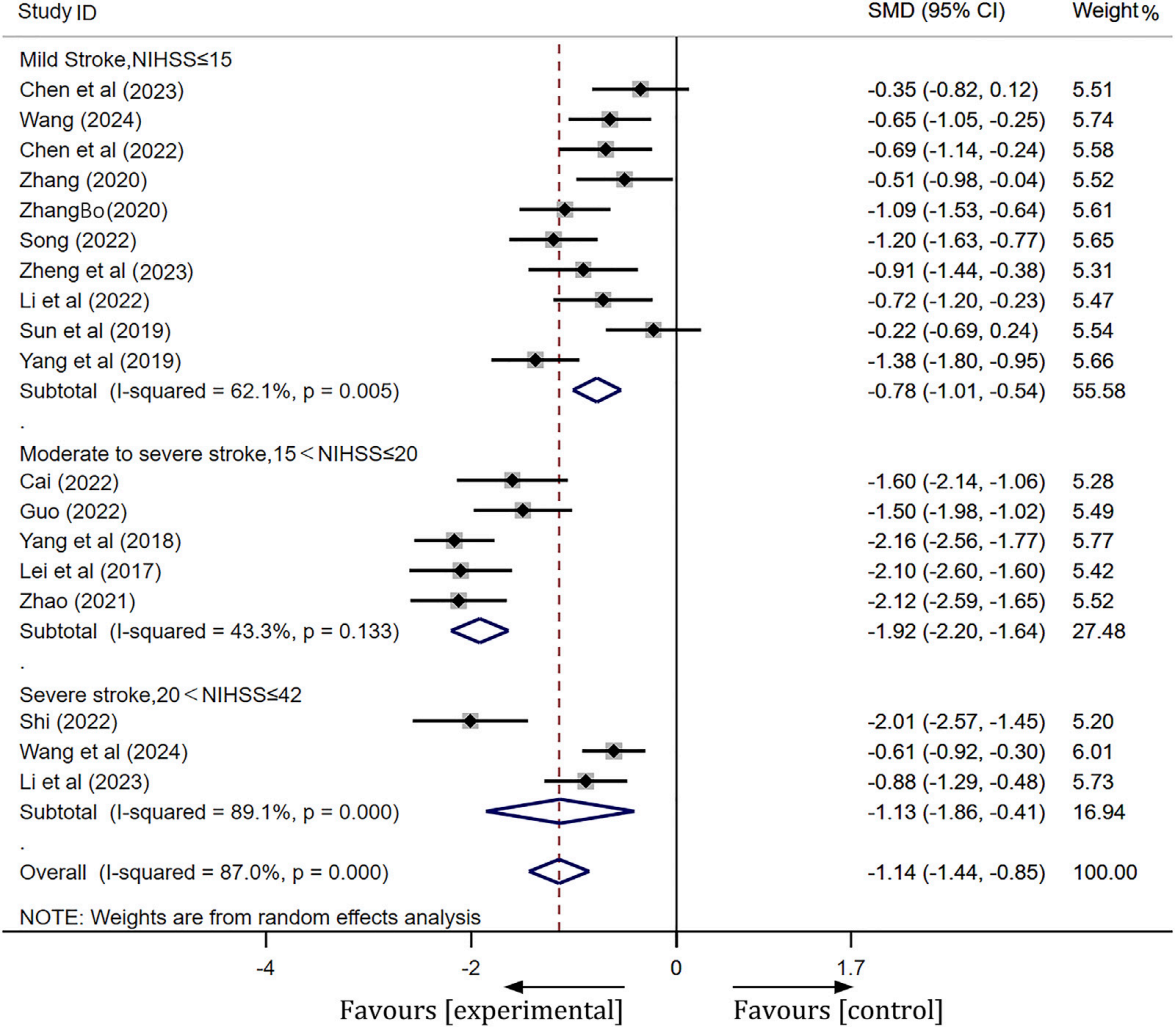
Forest plot of NIHSS scores after treatment.

A total of 19 studies were included in this analysis. Following heterogeneity testing, these studies exhibited high levels of heterogeneity (I^2^ = 94.5%, *p* < 0.01) (Supplementary Figure S2). Subsequently, sensitivity analysis revealed that Liu’s study (Liu, 2019) significantly influenced the meta-analysis results (Supplementary Figure S3). After excluding Liu’s study, we categorized patients into three subgroups based on their NIHSS scores prior to treatment: moderate stroke (NIHSS ≤15), moderate-severe stroke (15 < NIHSS ≤20), and severe stroke (20 < NIHSS ≤42). After conducting subgroup analysis and excluding outliers (Liu, 2019), heterogeneity was markedly reduced in the moderate stroke group (I^2^ = 62.1%, *p* < 0.01) and the moderate-severe stroke group (I^2^ = 43.3%, *p* = 0.13). A meta-analysis employing a random-effects model across three subgroups revealed statistically significant differences. Detailed meta-analysis results are as follows: for the mild stroke group, [SMD = −0.78, 95% CI(-1.01, −0.54), *p* < 0.01]; for the moderate-severe stroke group, [SMD = −1.92, 95% CI(-2.20, −1.64), *p* < 0.01]; and for the severe stroke group, [SMD = −1.13, 95% CI(-1.86, −0.41), *p* < 0.01]. These findings indicate that ginkgo terpene lactone preparations, when combined with antiplatelet drugs, is more effective in enhancing neurological function than the use of antiplatelet drugs alone. More details of the trials were presented in Figure 4.

#### Barthel index

**FIGURE 5:**
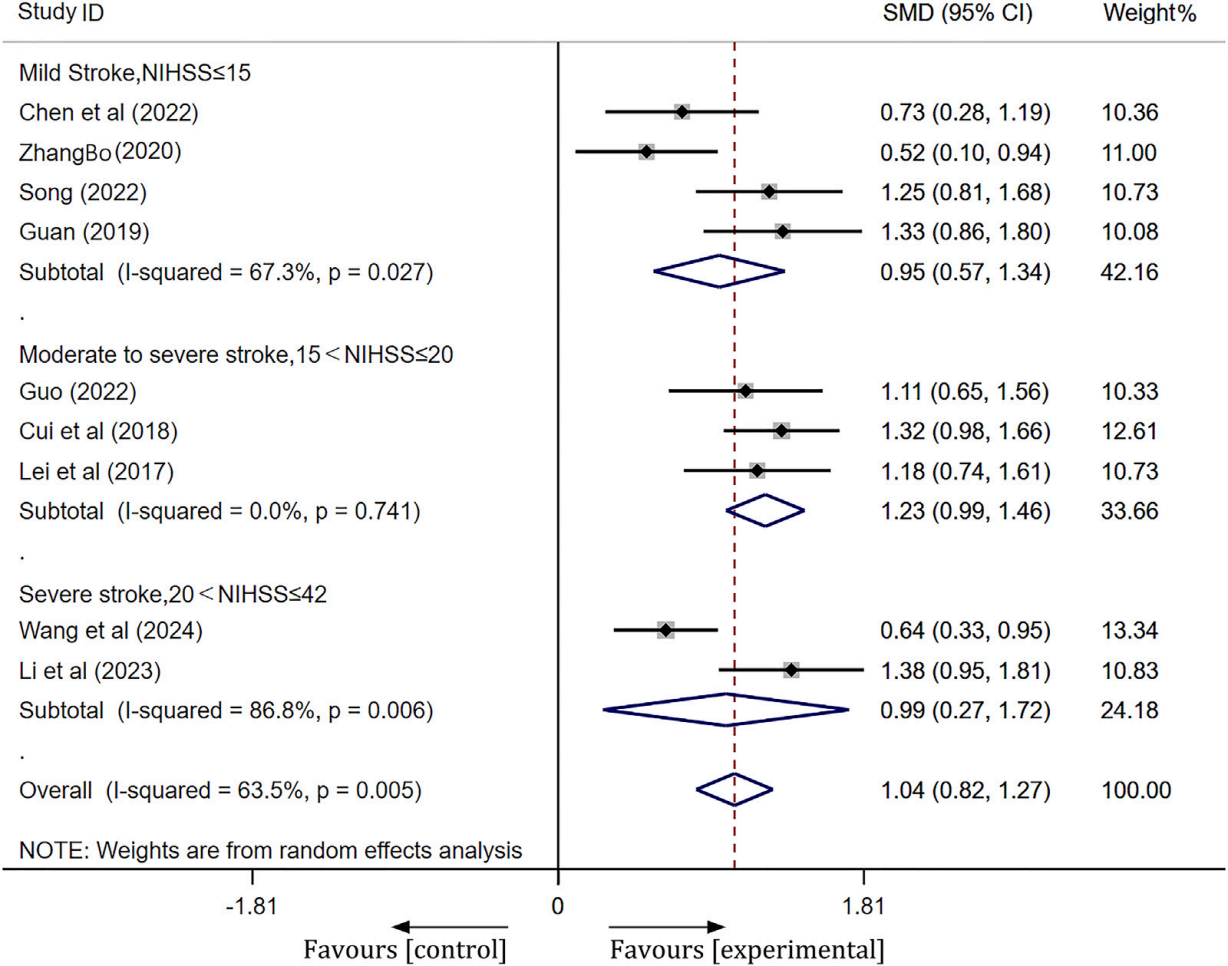
Forest plot of Barthel index after treatment.

A total of 10 studies were included. Following heterogeneity testing, these studies demonstrated a high level of heterogeneity (I^2^ = 80.2%, *p* < 0.01) (Supplementary Figure S4). Subsequently, sensitivity analysis identified Liu’s study (Liu, 2019) as the primary source of heterogeneity (Supplementary Figure S5). After excluding outliers (Liu, 2019), subgroup analysis based on pretreatment NIHSS scores revealed a significant reduction in heterogeneity. The results of the random effects model meta-analysis were as follows: for the mild stroke group [SMD = 0.95, 95% CI(0.57, 1.34), *p* < 0.001]; for the moderate-severe stroke group [SMD = 1.23, 95% CI(0.99, 1.46), *p* < 0.001]; and for the severe stroke group [SMD = 0.99, 95% CI(0.27, 1.72), *p =* 0.007]. The meta-analysis indicated that the Barthel index of the experimental group was significantly better than that of the control group, as illustrated in Figure 5.

#### mRS scores

**FIGURE 6:**
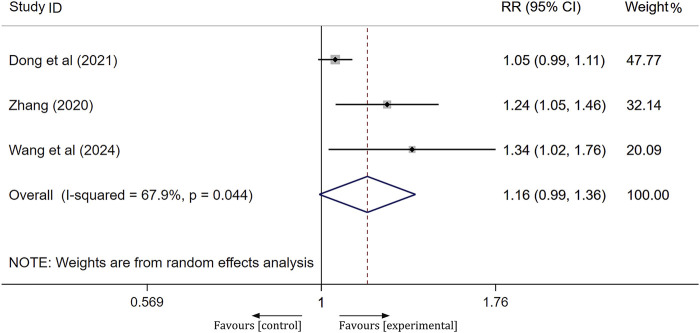
Forest plot of mRS score.

A total of three studies assessed the prognosis of patients following treatment using the mRS score, where a score of 0–2 indicates a good prognosis and a score of 3–6 indicates a poor prognosis. Significant heterogeneity was observed across the studies (I^2^ = 67.9%, *p* = 0.044); therefore, a random-effects model was employed to aggregate the effect sizes. The results of the meta-analysis indicated that the prognosis of the experimental group was not significantly different from that of the control group, with no notable difference between the groups [RR = 1.16, 95% CI(0.99, 1.36), *p* = 0.065], as shown in Figure 6.

#### Platelet function

**FIGURE 7:**
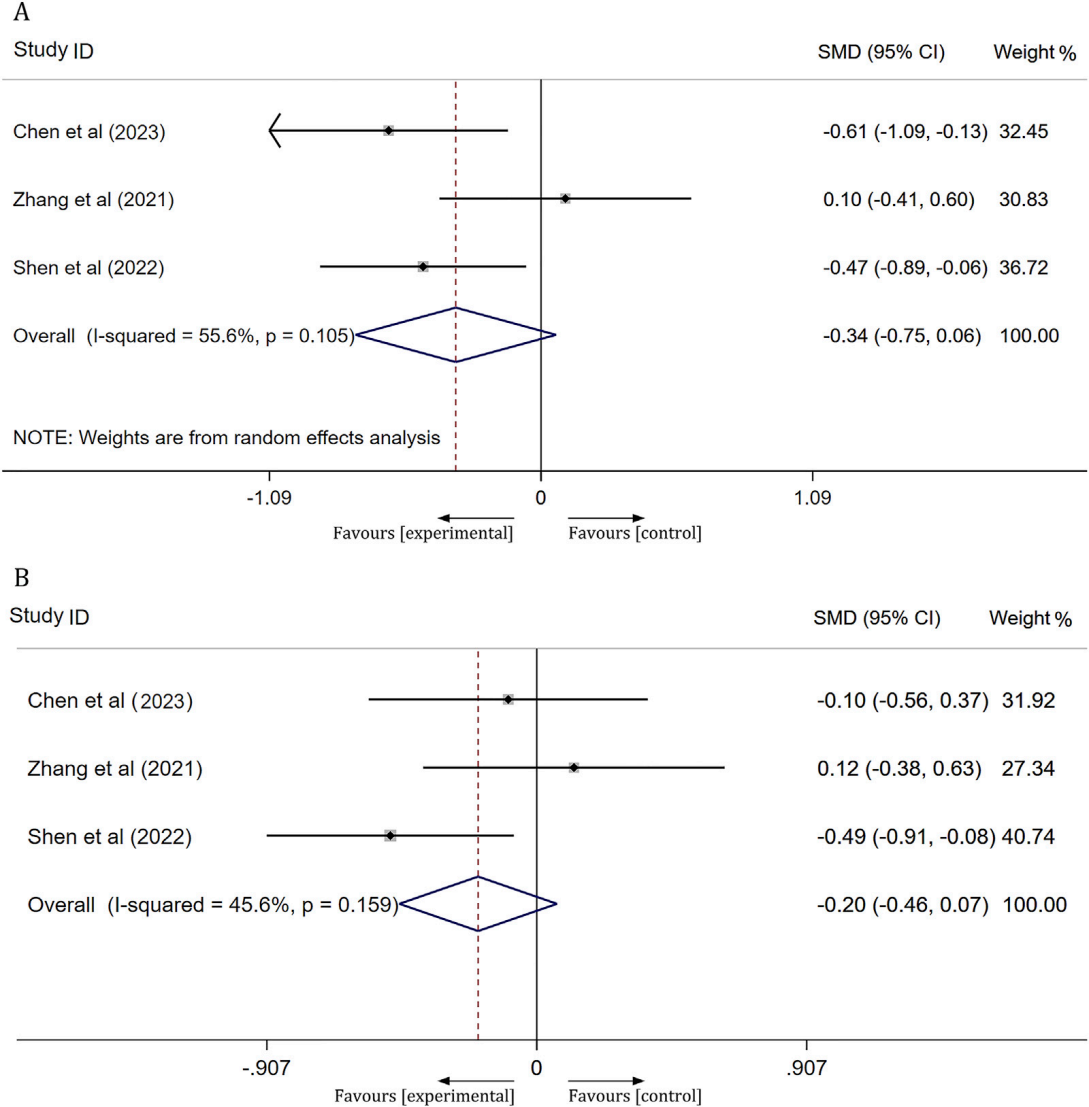
Forest plot of Platelet function. **(A)** AA-MAR; **(B)** ADP-MAR.

Three studies assessed platelet function in patients with ischemic stroke after 14 days of treatment, focusing on arachidonic acid induced maximal platelet aggregation rate (AA-MAR) and adenosine diphosphate induced maximal platelet aggregation rate (ADP-MAR). The results from a meta-analysis utilizing a random-effects model indicated no significant difference in AA-MAR between the experimental and control groups [SMD = -0.34, 95% CI(-0.75, 0.06), *p* = 0.095]. Similarly, a meta-analysis using a fixed-effects model revealed no significant difference in ADP-MAR between the experimental and control groups [SMD = −0.20, 95% CI(−0.46,0.07), *p* = 0.145]. For further details, refer to Figure 7.

#### Safety

**FIGURE 8:**
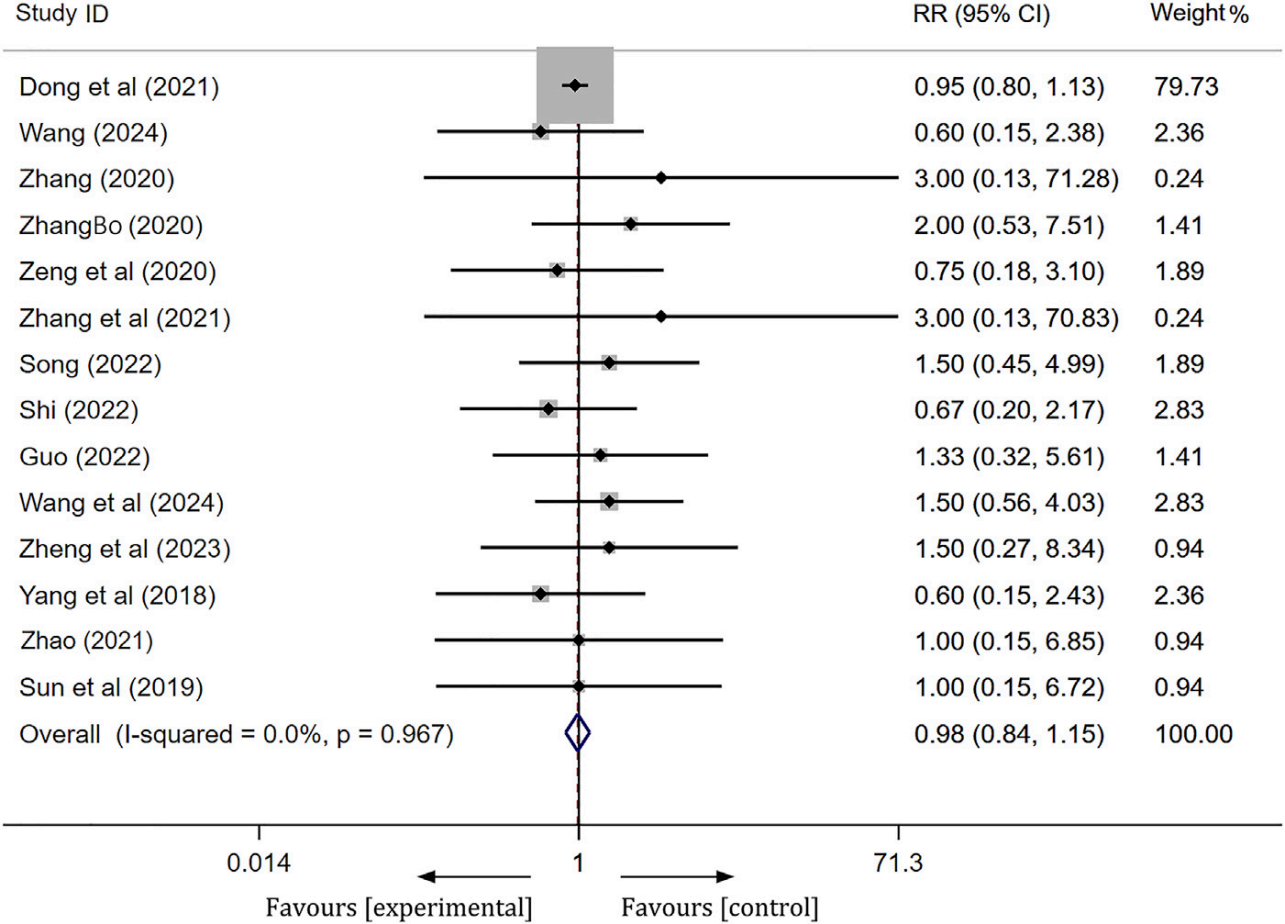
Forest plot of ADRs/ADEs.

Among the 27 studies included, three studies (Chen et al., 2023; Li et al., 2023; Cui et al., 2018) reported no significant serious ADRs associated with the treatment. Fourteen studies documented the occurrence of ADRs, with 206 patients in the experimental group and 213 patients in the control group experiencing such reactions. The clinical symptoms observed in both groups primarily included rash, nausea/vomiting, gastrointestinal discomfort, constipation, dizziness, palpitations, and abnormal liver and kidney function. Following the onset of these adverse reactions, patients in both groups demonstrated improvement or resolution of symptoms after receiving appropriate treatment. A meta-analysis employing a fixed effects model revealed no statistically significant difference in the incidence of ADRs between the experimental and control groups [RR = 0.98, 95% CI(0.84, 1.15), *p* = 0.84],as shown in Figure 8. This finding indicates that the addition of ginkgo terpene lactone preparations to the treatment regimen for ischemic stroke does not elevate the incidence of adverse events when compared to antiplatelet therapy alone. Furthermore, the remaining ten studies did not provide information regarding the occurrence of ADRs.

#### Publication bias

**FIGURE 9:**
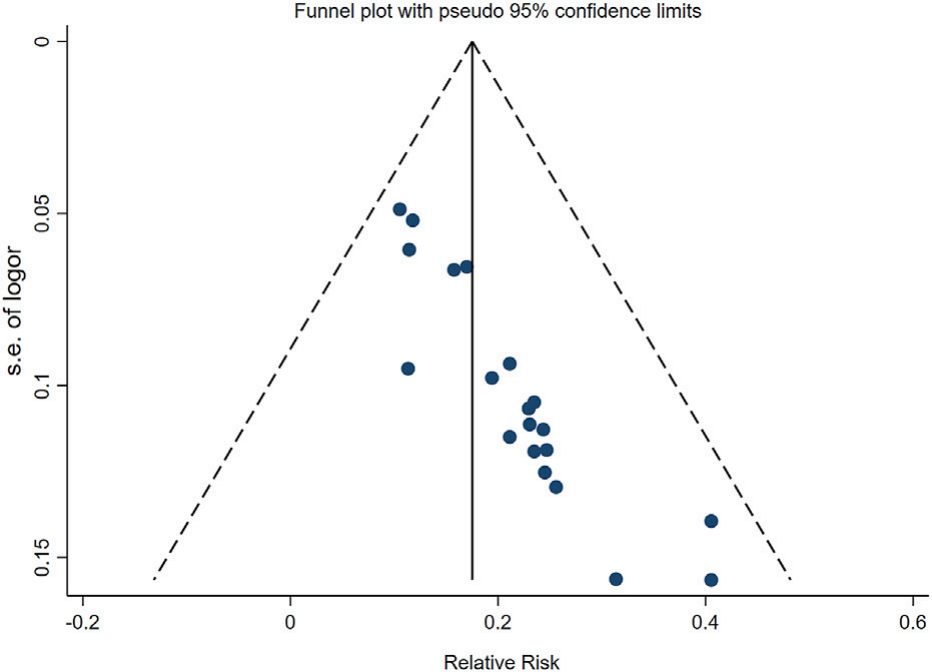
Funnel plot of publication bias.

We drawed a funnel plot for the clinical efficacy of treatment to evaluate publication bias. As illustrated in Figure 9, the funnel plot revealed an asymmetric distribution of the scatter on both sides of the invalid line, suggesting the presence of publication bias. Additionally, we conducted Egger’s test using Stata 17.0 software, and the results showed *p* < 0.05, further indicating the existence of publication bias.

## Discussion

This study included a total of 27 RCTs and conducted a meta-analysis to evaluate the efficacy and safety of ginkgo terpene lactone preparations in conjunction with conventional antiplatelet drugs for the treatment of ischemic stroke. The results indicated that the addition of ginkgo terpene lactone preparations to standard antiplatelet therapy enhances the overall clinical efficacy in patients with varying degrees of ischemic stroke, reduces the severity of neurological dysfunction, and improves activities of daily living. However, this combination regimen did not yield a significant improvement in the mRS scores and platelet function in patients with ischemic stroke when compared to antiplatelet therapy alone. Regarding safety, 14 studies explicitly reported side effects experienced by subjects during treatment, with the most common being dizziness and rash, followed by nausea, vomiting, mild abdominal pain, and other gastrointestinal symptoms. Overall, no major adverse events were reported in either group, and there was no statistically significant difference in the incidence of adverse reactions between the two treatment groups.

Traditional Chinese medicine (TCM) classifies ischemic stroke as a type of stroke, with phlegm-stasis blocking collateral syndrome being the most common variant. Ginkgo biloba has been utilized as a traditional Chinese medicinal plant since ancient times, primarily for its functions in promoting blood circulation, removing blood stasis, dredging collaterals, relieving pain, converging lung function, alleviating asthma, and dissipating turbidity while lowering lipid levels. Its ability to enhance blood circulation and eliminate blood stasis, along with its properties for dredging collaterals and dissipating turbidity, are significant reasons for the use of Ginkgo biloba in stroke treatment. Ginkgo terpene lactones are active components derived from the leaves of Ginkgo biloba, primarily consisting of diterpene and sesquiterpene structures (Geng et al., 2018). DGMI and ginkgolide injections are Ginkgo terpene lactone preparations that have received marketing approval, boasting high active ingredient content and minimal adverse effects. Furthermore, prior research has demonstrated that Ginkgo biloba terpene lactone preparations are linked to cerebral protection, a reduction in inflammatory responses, and enhanced microcirculation, making them beneficial in the prevention and treatment of cardiovascular and cerebrovascular diseases (Fang et al., 2015; Fang et al., 2010; Li et al., 2007; Chen M. et al., 2018; Fei et al., 2021). This study further corroborates that ginkgolide preparations can serve as adjuncts to antiplatelet therapy in patients with ischemic stroke. It is recommended that DGMI (25 mg, once daily) and ginkgolide injections (25 mg or 50 mg, once daily) be administered for a duration of 2 weeks. Nevertheless, in clinical practice, treatment strategies should still be tailored to the individual needs of each patient.

Our study also demonstrated that the addition of ginkgo terpene lactone preparations to conventional antiplatelet therapy did not significantly inhibit platelet aggregation induced by the arachidonic acid (AA) pathway or the adenosine diphosphate (ADP) pathway in patients. A multicenter randomized clinical trial (Dong et al., 2021) demonstrated that the effect of ginkgolide on ischemic stroke is associated with the platelet activating factor (PAF) pathway, but not with the ADP or thromboxane A2 (TxA2) pathways. Similarly, Han’s research (Han et al., 2023) found that neither AA-mediated nor ADP-mediated platelet reactivity, as measured by thromboelastography (TEG), differed significantly between DGMI-treated and untreated groups. Collectively, these studies suggest that the therapeutic benefits of ginkgo terpene lactone preparations may not be related to platelet aggregation induced by the AA or ADP pathways, which aligns with our findings. Furthermore, the results of the meta-analysis revealed no significant increase in the number of patients with good prognoses when ginkgo terpene lactone preparations was added to the treatment regimen compared to antiplatelet therapy alone. Our further analysis of the original literature suggested that this may be attributed to bias arising from the shorter follow-up duration after treatment in the study by Dong et al. (2021), and it may also be related to the limited number of RCTs included.

Currently, there are four systematic reviews (Zhao et al., 2022; Li et al., 2024; Zhang L. et al., 2021; Meng et al., 2021), both domestically and internationally, assessing the efficacy and safety of ginkgo terpene lactone preparations in conjunction with conventional Western medicine. Compared to their study, all ischemic stroke patients included in our research received standard antiplatelet therapy. Patients undergoing thrombolytic therapy or those receiving other neuroprotective agents, such as edaravone or citicoline, were excluded from our analysis. This exclusion was implemented to minimize the potential influence of thrombolytic therapy and similar interventions on the experimental outcomes. Furthermore, we strictly limited the duration of ginkgo terpene lactone preparations administration to 14 days within the experimental group, ensuring the generation of more rigorous and reliable study results. In instances of high heterogeneity, we conducted subgroup analyses based on the severity of ischemic stroke, followed by sensitivity analyses to identify the sources of heterogeneity among the indicators. To demonstrate the robustness of our findings, we also performed sensitivity analyses and assessed publication bias concerning the overall treatment response rate.

Despite the contributions of our study, several limitations should be acknowledged. First, we included 27 publications, all of which were in Chinese and English, potentially overlooking valuable studies published in other languages. Second, only three articles evaluated the effects of ginkgo terpene lactone preparations combined with antiplatelet drugs on platelet function and mRS scores in patients with ischemic stroke, this small sample size may introduce bias into the results. Finally, the majority of the RCTs included in this meta-analysis were small-scale studies conducted at single centers. Additionally, these studies often lacked clear allocation concealment and blinding during implementation, which increases the potential for bias. In future, a multicenter, large sample size, and double-blind placebo-controlled design is needed to verify the reliability of this meta-analysis.

## Data Availability

The original contributions presented in the study are included in the article/Supplementary Material, further inquiries can be directed to the corresponding authors.
