# Supplementary material for: The efficacy and safety of ginkgo terpene lactone preparations combined with antiplatelet aents in the treatment of ischemic stroke: a systematic review and meta-analysis
Source: Front Pharmacol. 2025 Mar 19;16:1554207. doi: 10.3389/fphar.2025.1554207 (PMC11961975; doi:10.3389/fphar.2025.1554207)
Supplement: Supplementary file 1 [file DataSheet1.docx]

Supplementary Material

# Supplementary Figures


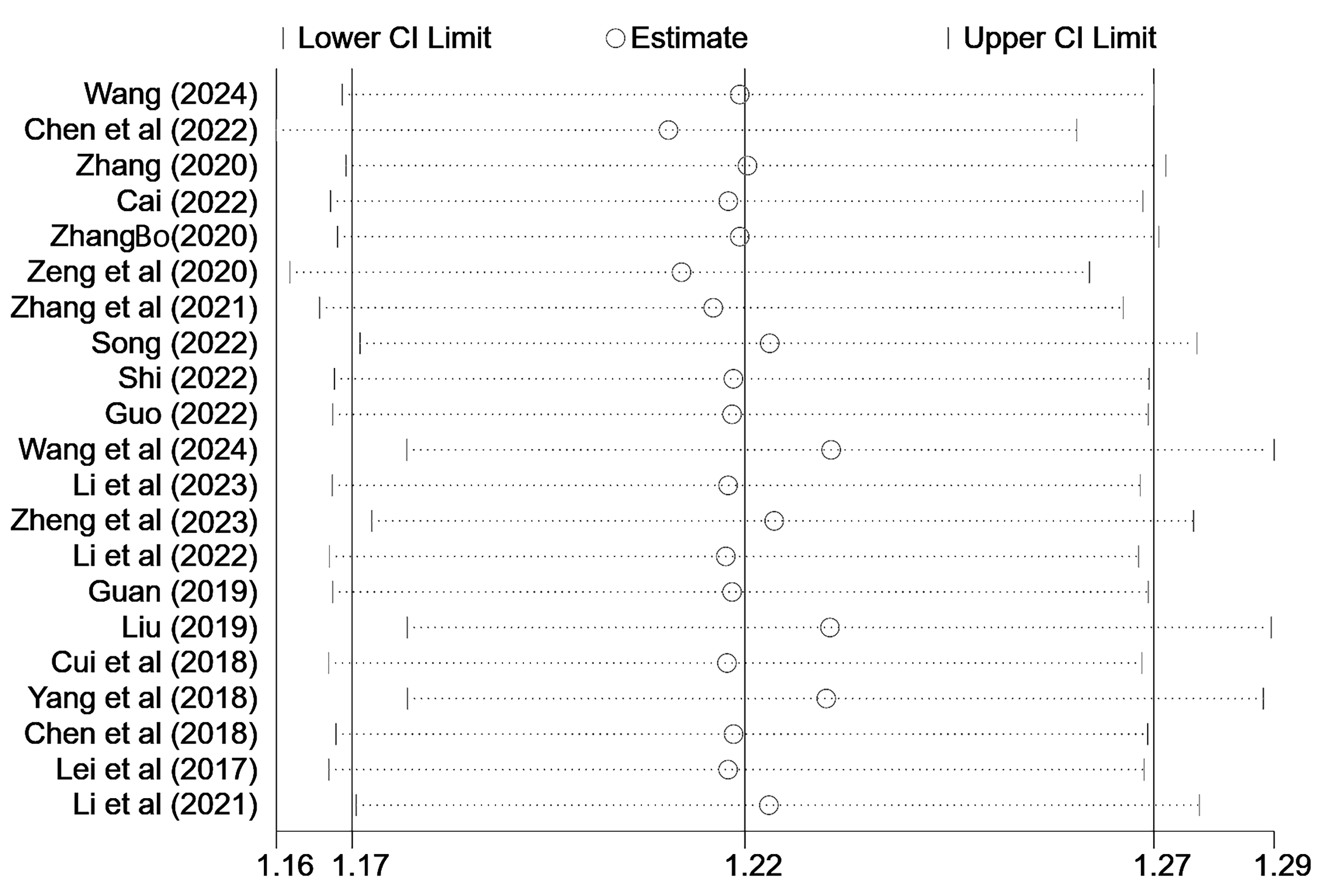


**Supplementary Figure 1.** Sensitivity analysis of clinical efficacy.

**
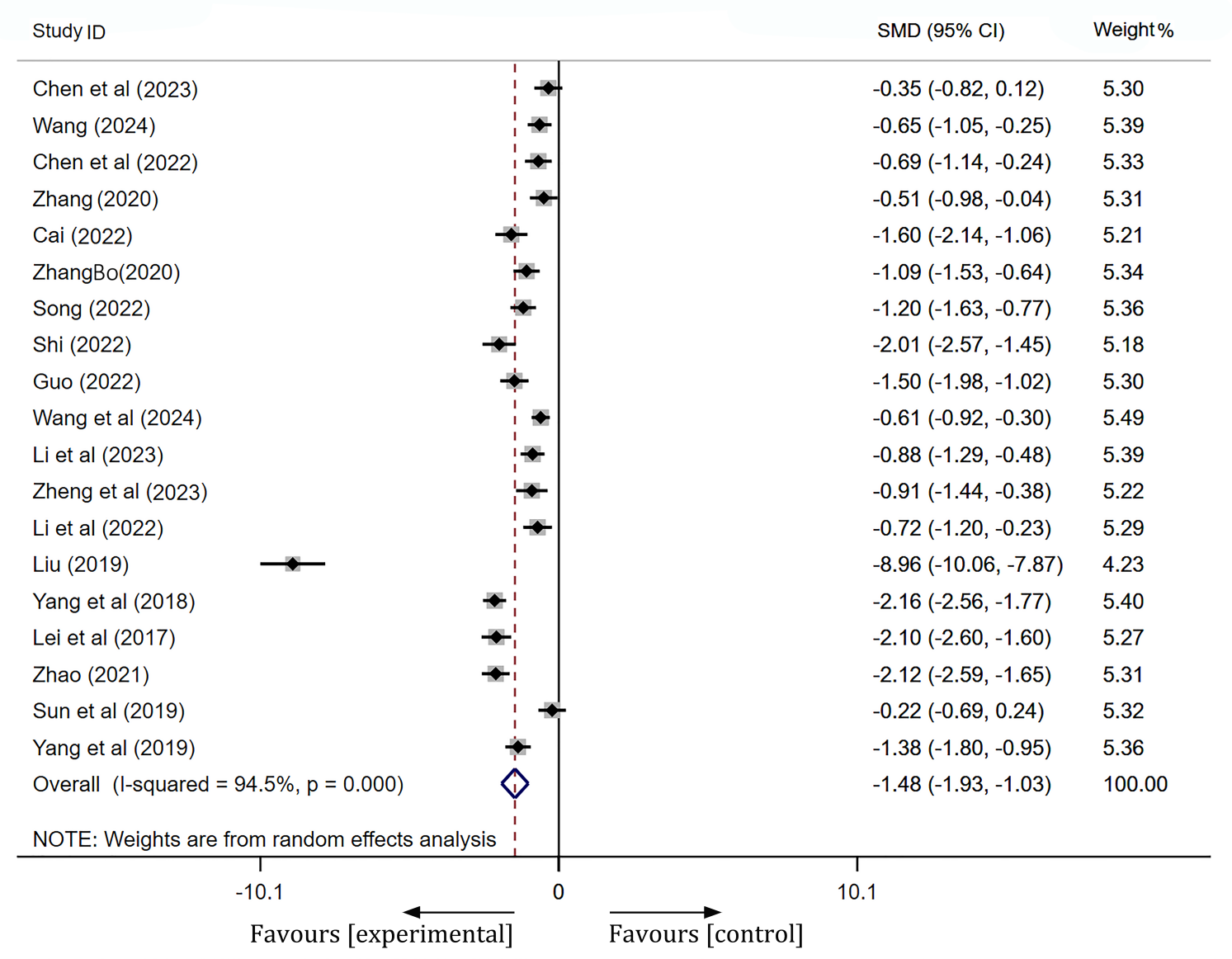
**

**Supplementary Figure 2. Forest plot of NIHSS scores after treatment (19 studies).**

**
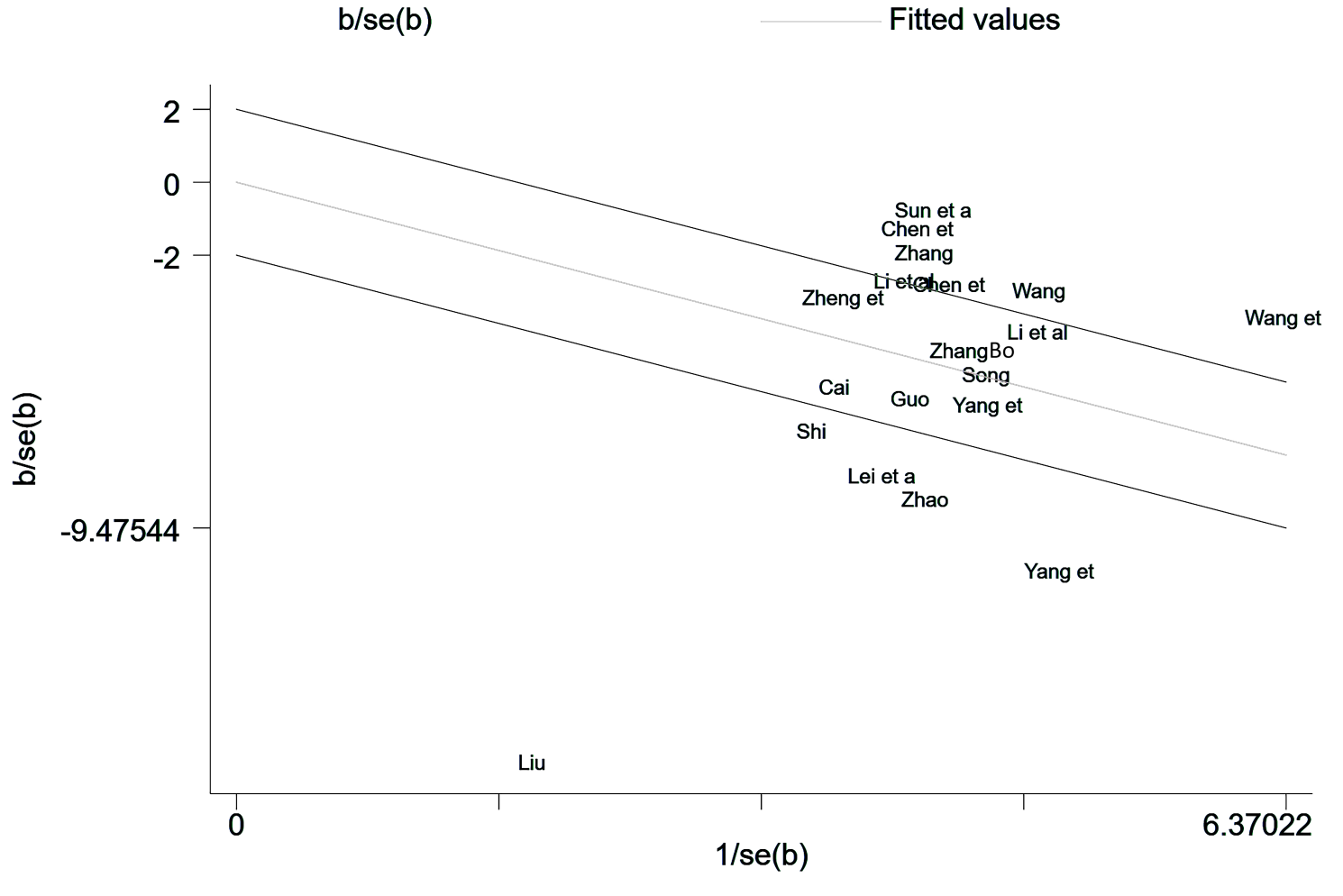
**

**Supplementary Figure 3. Sensitivity analysis in NIHSS score.**

**
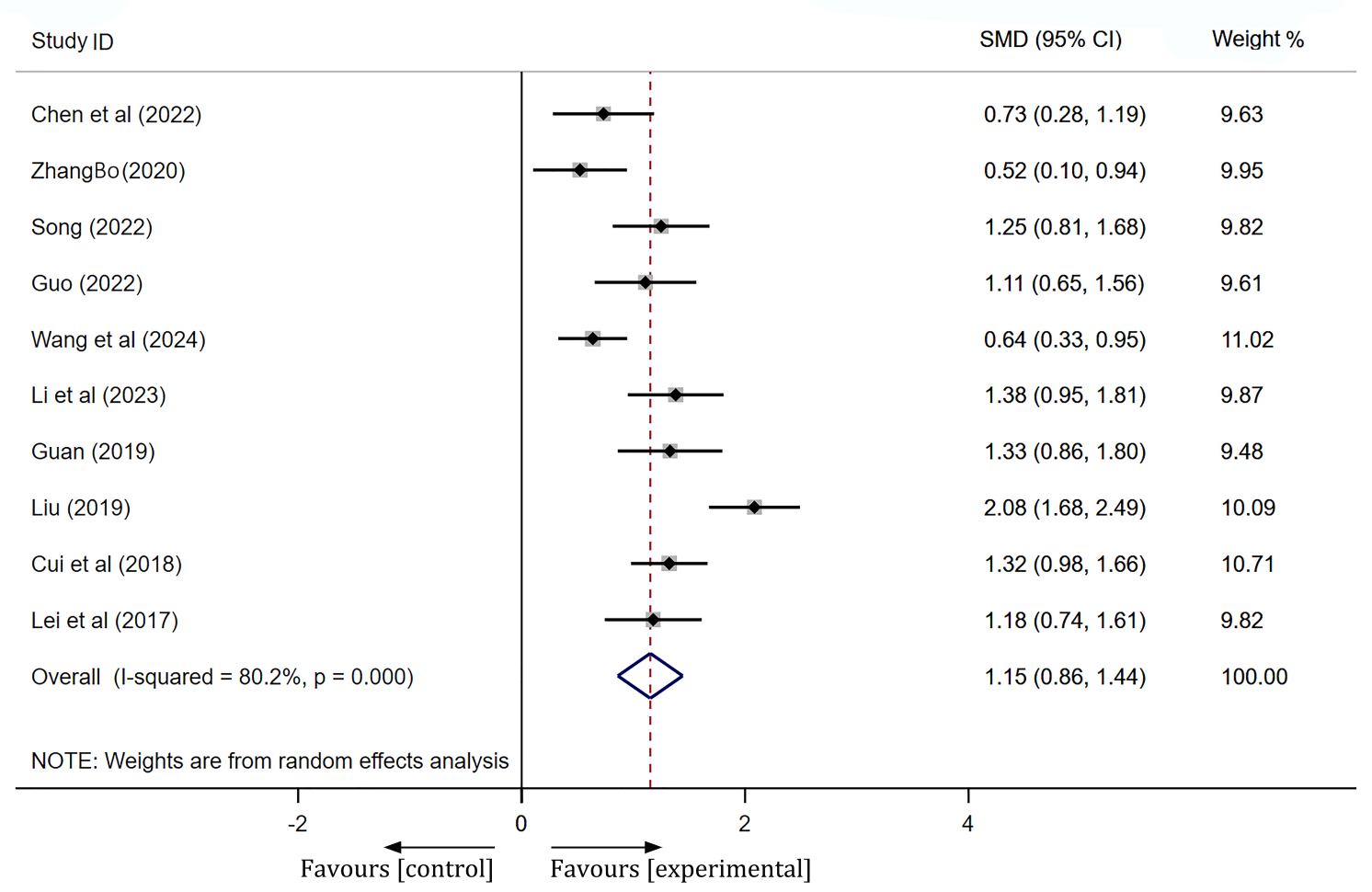
**

**Supplementary Figure 4. Forest plot of Barthel index after treatment (10 studies).**

**
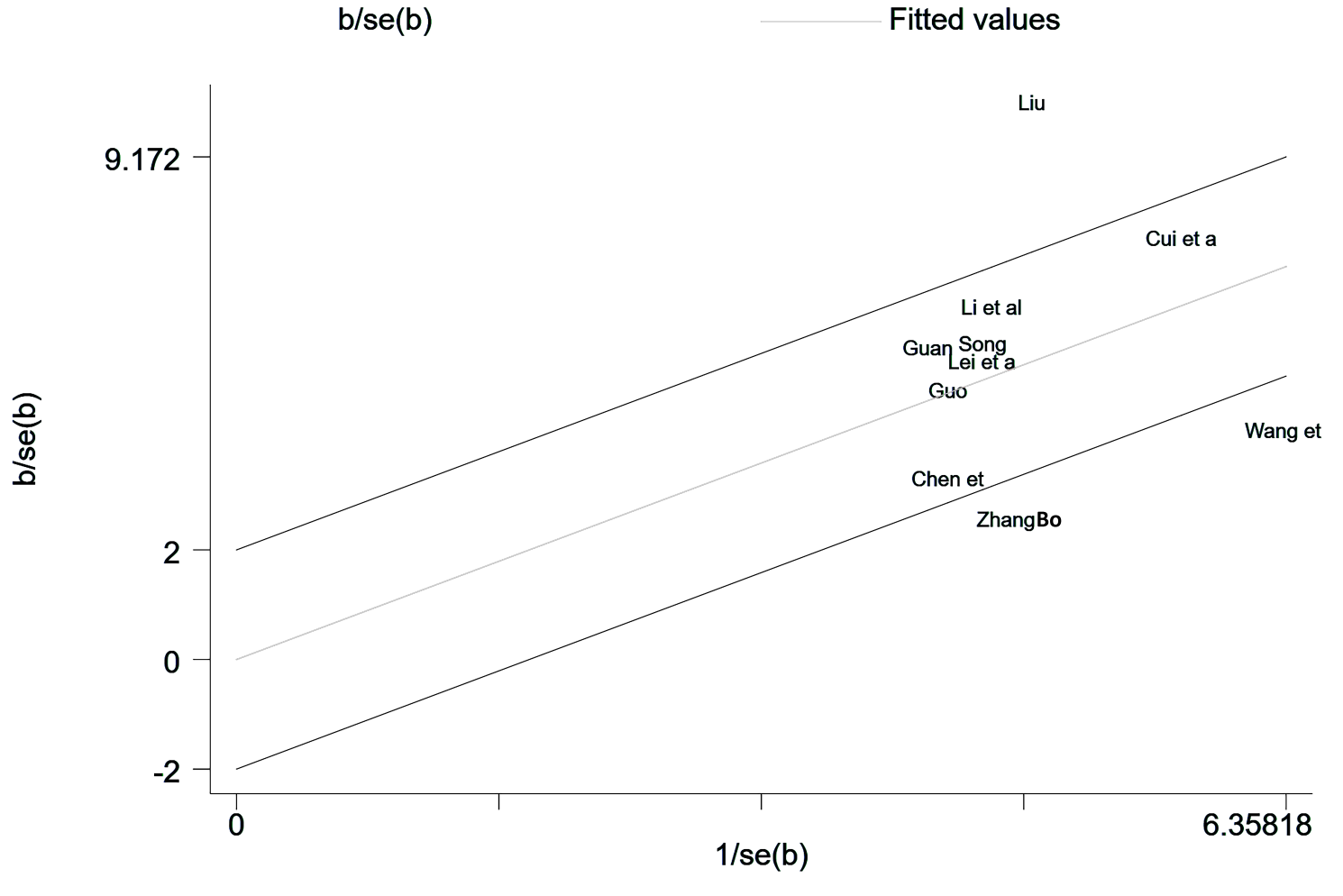
**

**Supplementary Figure 5. Sensitivity analysis in Barthel index.**
